# Serum ferritin as a crucial biomarker in the diagnosis and prognosis of intravenous immunoglobulin resistance and coronary artery lesions in Kawasaki disease: A systematic review and meta-analysis

**DOI:** 10.3389/fmed.2022.941739

**Published:** 2022-08-10

**Authors:** Huai Wen, Marady Hun, Mingyi Zhao, Phanna Han, Qingnan He

**Affiliations:** ^1^Department of Pediatrics, The Third Xiangya Hospital, Central South University, Changsha, China; ^2^Department of Ophthalmology, The Second Xiangya Hospital, Central South University, Changsha, China

**Keywords:** serum ferritin, Kawasaki disease, coronary artery lesions (CALs), intravenous immunoglobulin resistance, ferroptosis

## Abstract

**Background:**

Early identification and treatment are paramount for intravenous immunoglobulin (IVIG) resistance and coronary artery lesions (CALs) in patients with Kawasaki disease (KD). Unfortunately, there is no single crucial biomarker to identify these patients in a timely manner, which makes KD the most common cause of acquired heart disease in children in developed countries. Recently, many studies have focused on the association between serum ferritin (SF), IVIG resistance, and CALs in KD. We thus performed a systematic review and meta-analysis to ascertain the diagnostic and prognostic values of SF in predicting IVIG resistance and CALs in KD in the acute phase.

**Methods:**

The pooled sensitivity, specificity, positive likelihood ratio (PLR), negative likelihood ratio (NLR), diagnostic odds ratio (DOR), and area under the receiver operating characteristic curve (AUC) were extracted from the data to evaluate the SF levels in KD. The hazard ratios (HRs) of related risk factors and their corresponding 95% confidence intervals (CIs) were applied to compute the pooled assessments of the outcomes.

**Results:**

A total of 11 eligible articles were included in this meta-analysis, including twenty studies for diagnosis and five studies for prognosis. In terms of diagnostic values, SF could identify KD patients in the overall studies with a relatively high pooled sensitivity, specificity, PLR, NLR, DOR, and AUC of 0.76 (95% CI: 0.69–0.82), 0.82 (95% CI: 0.76–0.88), 4.33 (95% CI: 3.07–6.11), 0.29 (95% CI: 0.22–0.38), 15.0 (95% CI: 9.00–25.00), and 0.86 (95% CI: 0.83–0.89), respectively. In studies comparing KD patients and controls, there were a relatively high pooled sensitivity, specificity, PLR, NLR, DOR, and AUC of 0.79 (95% CI: 0.72–0.84), 0.84 (95% CI: 0.79–0.91), 4.61 (95% CI: 3.27–6.51), 0.26 (95% CI: 0.20–0.34), 20.82 (95% CI: 11.83–36.64), and 0.89 (95% CI: 0.86–0.91), respectively. For the prognostic values, we found poor survival outcomes based on KD patients (HR = 1.31, 95% CI: 1.07–1.59, *P* = 0.008).

**Conclusion:**

Our meta-analysis suggests that SF may be used as a workable and critical biomarker for the diagnosis and prognosis of IVIG resistance and CALs in patients with KD. We also propose that maintaining the dynamic balance between iron, SF, and ferroptosis will be an important therapeutic strategy to reduce the morbidity of CALs.

**Systematic review registration:**

[https://www.crd.york.ac.uk/prospero/], identifier [CRD42022279157].

## Introduction

Kawasaki disease (KD) is an acute systemic vasculitis with unknown etiology, which is the most common cause of pediatric acquired heart disease in developed countries and may result in long-term cardiac sequelae during adulthood (1). Moreover, according to the recent global epidemiology of vasculitis, Kawasaki disease occurs most frequently in East Asia, especially Japan, South Korea, and China, with a relatively equal distribution elsewhere (2). Virtually all deaths of patients with KD result from coronary artery lesions (CALs) (3). Many cases of fatal and non-fatal myocardial infarction (MI) in young adults have been attributed to “missed” KD in childhood (4). KD and KD-related cardiac sequelae create an enormous burden on individuals, families, and society. However, the diagnosis of KD mainly depends on patients’ clinical symptoms, which directly lead to a delay in the diagnosis and treatment of many children with incomplete KD (i.e., patients who do not have sufficient principal clinical findings) and increase the incidence of CALs. Although the timely initiation of intravenous immunoglobulin (IVIG) has been found to reduce the prevalence of CALs to approximately 4%, the incidence of CALs in KD remains high, and 10–20% of patients are resistant to initial IVIG treatment (1). Many studies have proven that patients who are resistant to initial IVIG are at increased risk of developing coronary artery abnormalities (5, 6). Thus, exploring potential biomarkers to diagnose or predict IVIG resistance and CALs in patients with KD has become a very fascinating area, but the predictive values of recognizing biomarkers, for example, C-reactive protein (CRP) or erythrocyte sedimentation rate (ESR), are partly limited (1, 7).

Elemental iron is one of the most plentiful and widely used metals on Earth (8). As an important trace element, it plays an essential role in a wide range of key biological processes in all living organisms, including DNA synthesis, energy production (oxygen transport and ATP production), and immune function (9). These biological functions of iron are mainly dependent on its ability to reversibly gain or lose a single electron to participate in oxidation–reduction reactions, which also catalyze the generation of reactive oxygen intermediates (ROIs) (10). It is possible that the disruption of iron homeostasis tends to cause cell death and human diseases because it serves as a mediator to promote the production of reactive oxygen species (ROS); thus, iron homeostasis is tightly controlled by a series of finely tuned, complex mechanisms (11).

Of these elaborate regulatory mechanisms, ferritin is one of the most fascinating. Ferritin is a hollow iron storage protein that consists of a protein shell and an iron core. The protein shell is composed of 24 highly symmetrical subunits of ferritin heavy chain (FTH) and ferritin light chain (FTL), and in the cavity of the protein shell, ferritin can bind different amounts of Fe^3+^ in a redox-inactive form (12, 13). In addition to iron storage, ferritin functions as a protective agent preventing iron overload and decreasing oxidative stress (OS) (14, 15). Growing evidence has indicated that ferritin acts as a switch in the dynamic balance of iron, that is, it is synthesized in response to high cellular iron levels and is degraded to release iron when iron demand is increased (14, 15). Unfortunately, free excess iron may be an OS source that leads to cell damage if it is not correctly stored in ferritin cores. Specifically, excessive free iron induces OS through the Fenton reaction, thereby activating cell ferroptosis by the iron-induced accumulation of lipid peroxides and ROS (16–18). Ferroptosis, characterized by iron-dependent OS and lipid peroxidation, is another new mechanism underlying iron-induced cell death, which contributes to the pathophysiology of various diseases (17, 19).

Undoubtedly, maintaining homeostasis between iron availability and iron storage and steering ferroptosis orientation are extremely important to ensure normal physiological function and improve the outcome of diseases, and SF plays a pivotal role in equilibrating holistic homeostasis. Elevated ferritin concentration is a marker of high levels of stored iron. The detection of ferritin concentration is not only an important indicator to diagnose diseases with iron overload or iron deficiency but also a marker of inflammatory conditions or autoimmune disorders (18). Therefore, an increasing number of scholars have focused on FS as a diagnostic or prognostic biomarker to distinguish targeted diseases early and in a timely manner and evaluate the major complications (20, 21). Given this compelling rationale, a series of clinical trials have assessed the association between SF and KD, IVIG resistance, and CALs. Recognizing that individual studies might not be able to provide sufficient data on their own to affect practice, we sought to objectively assess the diagnostic or prognostic value of SF as a crucial biomarker in IVIG resistance and CALs in KD. We therefore performed a meta-analysis to establish the relationship between SF and KD, IVIG resistance, and CALs.

## Materials and methods

### Study protocol

We performed this systematic review and meta-analysis according to the PRISMA guidelines (22, 23). The meta-analysis with systematic review described herein has been accepted by PROSPERO, an online international prospective register of systematic reviews curated by the National Institute for Health Research (PROSPERO number: CRD42022279157). The population, intervention, comparison, and outcome worksheet are shown in Table 1.

**TABLE 1:** Participants, Intervention, Comparator, Outcomes, and Study design (PICOS) criteria for inclusion in the systematic review and meta-analysis.

Acronym	Definition	Application of the criteria
P	Population	Pediatric patients who had been diagnosed with KD
I	Intervention	SF was measured in all KD included patients
C	Comparison	Our study was evaluated the comparison between the included studies of the KD and all controls in SF levels
O	Outcome	The outcomes were the incidence and the risk factor of SF levels on KD
S	Study designs	Prospective and retrospective studies; case studies (*N* ≥ 20)

KD, Kawasaki disease; SF, serum ferritin.

### Search strategy

On 30 September 2021, PubMed, Web of Science, the Cochrane Central Register of Controlled Trials, Embase, and China National Knowledge Infrastructure (CNKI) databases were searched by using the following keywords to retrieve literature: (”mucocutaneous lymph node syndrome or Kawasaki syndrome or lymph node syndrome, mucocutaneous or Kawasaki disease”) and (”ferritins or ferritin or isoferritin or basic isoferritin or isoferritin, basic”). We considered all potentially eligible studies for review, irrespective of the primary outcome or language. We also performed a manual search using the reference lists of key articles published in English.

### Study selection criteria

Studies were enrolled in our meta-analysis based on the following criteria: (1) the diagnosis of Kawasaki disease was based on KD diagnostic criteria; (2) the studies were case–control, retrospective, or prospective studies with sample sizes of at least 20 cases and investigated the relationship between SF and KD; (3) the studies provided adequate information to build up true positives (TPs), true negatives (TNs), false positives (FPs), and false negatives (FNs) for diagnostic meta-analysis, and assessed hazard ratios (HRs) or odds ratios (ORs) and 95% confidence intervals (95% CI) for prognostic meta-analysis; and (4) the studies included KD patients with non-CALs vs. CALs and IVIG-responsive vs. IVIG-resistant subjects related to SF.

The following studies were excluded: (1) reviews, meta-analyses, meeting abstracts, proceedings papers, case reports, case series, editorials, letters, animal articles, and conference abstracts; (2) studies with no quantitative data or incomplete or unavailable data for SF levels; (3) duplicate publications; and (4) studies with incorrect statistical methods, deficiencies in the necessary information of TPs, TNs, FPs, and FNs for diagnostic meta-analysis, and contradictions in the process.

### Data extraction and quality assessment

Data extraction and quality assessment were incorporated into the exclusion and inclusion criteria by three authors (Marady Hun, Huai Wen, and Mingyi Zhao) independently: (1) the initial results were screened for titles, first author, year of publication, abstracts, study population, sample size, and study type; (2) TP, TN, FP, FN, area under the curve (AUC), and cutoff values were extracted for diagnostic meta-analysis, and related outcomes or risk factors for SF in HRs or ORs and 95% CIs were extracted for prognostic meta-analysis; (3) the self-designed data extraction table was used for included studies; and (4) the Quality Assessment of Diagnostic Accuracy Studies 2 (QUADAS2) was utilized for diagnostic meta-analysis (24), and the Newcastle–Ottawa Scale (NOS) was utilized for prognostic meta-analysis (25) to assess the quality of the included literature. If there were conflicts in the quality evaluation procedure, the consensus was extended through debate by the fourth investigator (Supplementary Figure 1 and Supplementary Table 1).

### Statistical analysis

We study used ReviewManager (RevMan) version 5.4^1^, Meta-DiSc 1.4^2^, and STATA 15.0 software^3^ to conduct statistical analysis of the included records.

For the diagnostic meta-analysis, to obtain accurate diagnostic components of SF for KD, including the pooled diagnostic odds ratio (DOR), sensitivity, specificity, positive likelihood ratio (PLR), negative likelihood ratio (NLR), summary receiver operator characteristic (SROC) curve, and AUC, we calculated TP, FP, FN, and TN values by using sensitivity (SEN) = [TP/(FN + TP)], specificity (SPE) = [TN/(FP + TN)], and diagnostic odds ratio (DOR) = [(1-SPE)/SPE]. A bivariate boxplot was performed to distinguish heterogeneity. The potential publication bias for the prognostic meta-analysis was computed by the ES (95% CI) value for each inclusion logarithm OR (log^OR^) value as the productive value indicator and was identified by the evaluated variance of the log^OR^. The generic inverse variance process was used for weighting, and a *P*-value < 0.05 was considered a statistically significant difference between the groups or subgroups. Forest plots were designed for related factors. Cochran’s *Q* test and the *I*^2^ value were used to estimate the heterogeneity for each selected study; *I*^2^ > 50% indicated the appearance of heterogeneity. The random-effects model was used for *I*^2^ > 50%; otherwise, the fixed-effects model was utilized (26). The funnel plot, Egger’s test, and Begg’s test were applied to determine the publication bias. A *P*-value < 0.05 was considered statistically significant (27).

## Results

**FIGURE 1:**
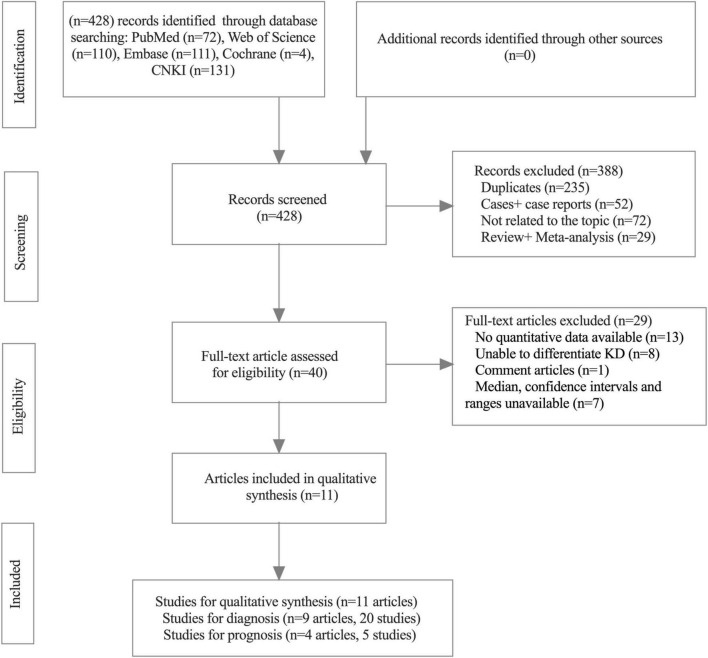
Flow diagram of study selection process.

The flow diagram utilized to illustrate the process of inclusion and exclusion (PRISMA statement) is shown in Figure 1. A total of 428 related records for SF and KD were initially recognized from PubMed (72 records), Web of Science (110 records), Cochrane Library (4 records), Embase (111 records), and CNKI (131 records) databases updated through 30September 2021. Of these, 338 records were excluded; 235 were duplicates, 29 were reviews and meta-analyses, 52 were cases and case reports, and 72 were not related to the topic. After the full-text articles of the remaining 40 records were assessed for eligibility, 29 records were excluded (the reasons for exclusion are detailed in Figure 1); ultimately, 11 records (nine records (20, 21, 28–34) for diagnostic meta-analysis and four records (33–36) for prognostic meta-analysis) were included in this meta-analysis. Of these 11 studies, seven were from China, two were from Korea, and two were from Japan (the quality of all enrolled studies for diagnostic meta-analysis is presented in Supplementary Figure 1). For diagnostic assessment, 20 studies from nine articles were enrolled in our meta-analysis and were separated into three comparable groups: (1) KD vs. controls (including fever, healthy, and s-JIA (systemic juvenile idiopathic arthritis) groups), 11 studies from six articles (totaling 1023 KD cases and 661 controls); (2) KD-CAL vs. KD-non-CAL, five studies from five articles (totaling 152 KD-CAL cases and 716 KD-non-CAL cases); and (3) KD-IVIG resistance vs. KD-IVIG responders, four studies from four articles (totaling 151 KD-IVIG resistance and 570 KD-IVIG responders). For prognostic assessment, a total of 894 patients were included from five studies (four articles) relevant to SF and KD (the quality of all enrolled studies for prognostic meta-analysis is presented in Supplementary Table 1). Among the enrolled studies in the diagnostic meta-analysis, 19 were retrospective trials, and one was a prospective trial; in the prognostic meta-analysis, all five were retrospective trials. The baseline characteristics of all included studies are summarized in Table 2 for the diagnostic meta-analysis and Table 3 for the prognostic meta-analysis.

**TABLE 2:** Characteristics of studies in diagnostic meta-analysis.

Study	Years	Country	Design	Diagnostic criteria	Sample size	Mean age (month)	Mean ferritins levels (ng/ml)	Cut-off of ferritins (ng/ml)	TP	FP	FN	TN	AUC	Sensitivity	Specificity
**KD patients vs. controls (including fever, healthy, and s-JIA groups)**															
Guo et al. (28)	2021a	China	RCS	AHA	KD vs. Fever: 60 vs. 60	KD vs. Fever: 33.84 vs. 33.96	KD vs. Fever: 192.75 ± 76.98 vs. 117.15 ± 51.61	142.45	46	16	14	44	0.792	76.70%	73.30%
Guo et al. (28)	2021b	China	RCS	AHA	KD vs. Healthy: 60 vs. 60	KD vs. Healthy: 33.84 vs. 39.00	KD vs. Healthy: 192.75 ± 76.98 vs. 72.21 ± 21.57	142.45	46	16	14	44	0.792	76.70%	73.30%
Kim et al. (21)	2021	Korea	PCS	NA	KD vs. Fever:77 vs. 32	KD vs. Fever:22.8 vs. 25.2	KD vs. Fever:188.8 (25.5–750.5) vs. 106.8 (11.1–632.2)	120.80	63	1	14	31	0.83	81.82%	95.76%
Pan et al. (29)	2019a	China	RCS	JKDRC	KD vs. s-JIA: 53 vs. 53	KD vs. s-JIA: 42 ± 7.2 vs. 43.2 ± 6	NA	254.7	38	7	15	46	0.83	72.00%	87.5%
Pan et al. (29)	2019b	China	RCS	JKDRC	KD vs. Healthy: 53 vs. 53	KD vs. Healthy: 42 ± 7.2 vs. 39.6 ± 4.8	NA	254.7	38	7	15	46	0.83	72.00%	87.5%
Wen et al. (31)	2018a	China	RCS	AHA	KD vs. Healthy: 108 vs. 30	KD vs. Healthy: (2–137) vs. (4–120)	KD vs. Healthy: 227 ± 238 vs. 72 ± 101	133.35	76	10	32	20	0.793	70.10%	66.70%
Wang et al. (32)	2016a	China	RCS	JKDRC	KD vs. s-JIA: 96 vs. 73	KD vs. s-JIA: 77.04 vs. 78.96	KD vs. s-JIA: 232.35 ± 155.95 vs. 1017.66 ± 584.18	239.5	82	17	14	56	NA	85.40%	76.50%
Wang et al. (32)	2016b	China	RCS	JKDRC	KD vs. s-JIA: 96 vs. 73	KD vs. s-JIA: 96 vs. 73	KD vs. s-JIA: 96 vs. 73	292.5	76	11	20	62	NA	79.20%	85.20%
Wang et al. (32)	2016c	China	RCS	JKDRC	KD vs. s-JIA: 96 vs. 73	KD vs. s-JIA: 96 vs. 73	KD vs. s-JIA: 96 vs. 73	385.5	74	6	22	67	NA	77.10%	91.40%
Wang et al. (32)	2016d	China	RCS	JKDRC	KD vs. s-JIA: 96 vs. 73	KD vs. s-JIA: 96 vs. 73	KD vs. s-JIA: 96 vs. 73	929.5	58	1	38	72	NA	60.40%	98.80%
Mao et al. (33)	2016a	Japan	RCS	AHA	KD vs. s-JIA: 228 vs. 81	KD vs. s-JIA: 24 (1.2-168) vs. 84 (7.2-312)	KD vs. s-JIA: 147.5 (14–2,376) vs. 1189 (63–68,310)	369.6	215	14	13	67	0.939	94.30%	82.70%
**CALs vs. non-CALs**															
Guo et al. (28)	2021c	China	RCS	AHA	CALs vs. non-CALs: 6 vs. 54	CALs vs. non-CALs: 29.4 vs. 25.44	CALs vs. non-CALs: 235.48 ± 95.71 vs. 188.01 ± 74.18	NA	5	14	1	40	NA	76.70%	73.30%
Kong et al. (30)	2019a	China	RCS	AHA	CALs vs. non-CALs: 48 vs. 252	CALs vs. non-CALs: 19 (9–33) vs. 25 (14–42)	CALs vs. non-CALs: 146.9 (105.6–217.4) vs. 152.6 (111.7–208.9)	NA	21	28	27	224	NA	43.00%	88.80%
Kim et al. (34)	2019	Korea	RCS	AHA	CALs vs. non-CALs: 55 vs. 118	CALs vs. non-CALs: 36 (18–63) vs. 29 (14–55)	CALs vs. non-CALs: 69.9 (47.4–112.4) vs. 24.1 (19.7–28.5)	30.6	45	5	10	113	0.907	81.82%	95.76%
Wen et al. (31)	2018b	China	RCS	AHA	CALs vs. non-CALs: 31 vs. 77	NA	CALs vs. non-CALs: 340 ± 405 vs. 183 ± 99	160.2	23	37	8	40	NA	73.70%	52.10%
Mao et al. (33)	2016b	Japan	RCS	AHA	CALs vs. non-CALs: 12 vs. 215	NA	NA	NA	8	99	4	116	NA	62.70%	54.00%
**IVIG resistance vs. IVIG responders**															
Kong et al. (30)	2019b	China	RCS	AHA	IVIG-resistance vs. IVIG- responders: 29 vs. 271	IVIG-resistance vs. IVIG- responders: 25 (15–47) vs. 24 (13–41)	IVIG-resistance vs. IVIG- responders: 198.6 (129.7–411.6) vs. 146.6 (107.2–205.5)	269.7	12	30	17	241	0.663	43.00%	88.80%
Wen et al. (31)	2018c	China	RCS	AHA	IVIG-resistance vs. IVIG- responders: 27 vs. 81	NA	IVIG-resistance vs. IVIG- responders: 257 ± 287 vs. 215 ± 216	133.35	19	27	8	54	0.623	70.10%	66.70%
Mao et al. (33)	2016c	Japan	RCS	AHA	IVIG-resistance vs. IVIG- responders: 67 vs. 161	NA	NA	144.3	63	28	4	133	0.618	94.30%	82.70%
Yamamoto et al. (20)	2015	Japan	RCS	JKDRC	IVIG-resistance vs. IVIG- responders: 28 vs. 57	IVIG-resistance vs. IVIG- responders: 32 (5–112) vs. 29 (2–124)	IVIG-resistance vs. IVIG- responders: 214.9 (50.6–558.5) vs. 141.1 (34.5–428.4)	165	20	21	8	36	0.674	70.40%	63.20%

RCS, retrospective cohort study; PCS, prospective cohort study; TP, true positives; FP, false positives; FN, false negatives; TN, true negatives; AUC, area under the receiver operating characteristic curve; KD, Kawasaki disease; s-JIA, systemic juvenile idiopathic arthritis; AHA, American Heart Association; JKDRC, Japan Kawasaki Disease Research Committee; IVIG, intravenous immunoglobulin; CALs, coronary artery lesions; NA, not available.

**TABLE 3:** Characteristics of studies in prognostic meta-analysis.

Study	Years	Country	Design	Diagnostic criteria	Sample size	Mean age (month)	Mean ferritins levels (ng/ml)	IVIG dosage	Aspirin (mg/kg/day)	OR (95%CI)
Tan et al. (35)	2021	China	RCS	AHA	IVIG-resistance vs. IVIG- responder: 15 vs. 77	IVIG-resistance vs. IVIG- responder: 31.56 ± 5.28 vs. 32.88 ± 5.64	IVIG-resistance vs. IVIG- responder: 190.62 ± 9.54 vs. 136.52 ± 8.97	2 g/kg	High-dose: 30–50 Low-dose: 3–5	1.21 (1.05–1.40)
Peng et al. (36)	2020	China	RCS	AHA	IVIG-resistance vs. IVIG- responder: 31 vs. 142	IVIG-resistance vs. IVIG- responder: 19.5 (9.0–40.5) vs. 17.0 (8.0–39.0)	IVIG-resistance vs. IVIG- responder: 133 (83–263) vs. 210 (151–277)	2g/kg	High-dose: 30–50 Low-dose: 3–5	1.19 (1.08–1.32)
Mao et al.*^1^ (33)	2016a	Japan	RCS	AHA	IVIG-resistance vs. IVIG- responder: 67 vs. 161	NA	NA	2 g/kg	NA	1.98 (1.10–3.54)
Mao et al.*^2^(33)	2016b	Japan	RCS	AHA	IVIG-resistance vs. IVIG- responder: 67 vs. 161	NA	NA	2 g/kg	NA	4.86 (1.50-15.78)
Kim et al. (34)	2019	Korea	RCS	AHA	CALs vs. non-CALs: 55 vs. 118	CALs vs. non-CALs: 36 (18–63) vs. 29 (14–55)	CALs vs. non-CALs: 69.9 (47.4–112.4) vs. 24.1 (19.7–28.5)	NA	NA	0.95 (0.93–0.97)

*^1^OR and 95% CI value when patients not needing plasma exchange. *^2^OR and 95% CI value when patients needing plasma exchange. RCS, retrospective cohort study; AHA, American Heart Association; IVIG, intravenous immunoglobulin; CALs, coronary artery lesions; NA, not available.

### Diagnostic value of serum ferritin for Kawasaki disease

#### Study characteristics and quality assessment

The quality of the included studies is outlined in Supplementary Figure 1. Of the 20 included studies, nine articles (2,612 related KD patients with SF testing) (20, 21, 28–34) demonstrated a moderate to high quality of all diagnostic studies. The sample size of the included studies ranged from 27 to 271, and the studies were published from 2015 to 2021. The characteristic details of the included literature are shown in Figure 1. To improve the quality of all included studies, they were separated into three subgroups: (1) the first group included studies comparing SF values of KD patients and different controls (including fever, healthy, and s-JIA groups); (2) the second group included studies comparing SF values of endogenous KD patients with CAL vs. non-CAL; and (3) the third group included studies comparing SF values of endogenous KD IVIG responders vs. IVIG resistance. The baseline characteristics of all included studies are summarized in Table 2.

#### Data analysis

**FIGURE 2:**
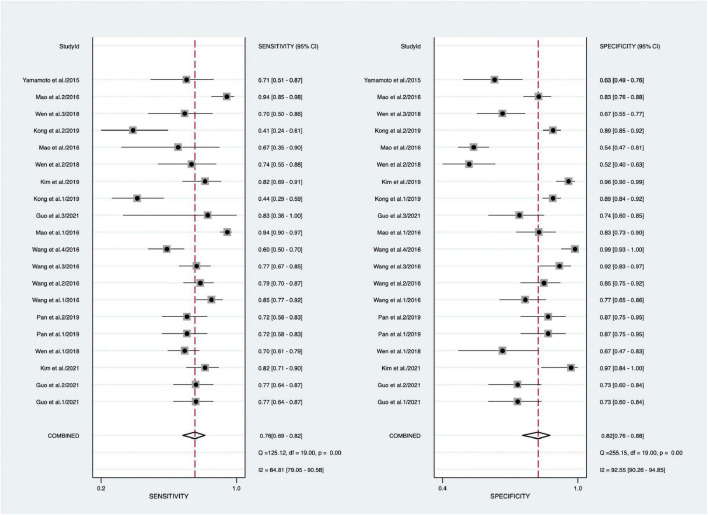
Forest plots of sensitivity and specificity of overall SF in the diagnosis of KD.

Forest plot results of related data from the 20 studies (nine articles) (20, 21, 28–34) on the sensitivity and specificity of overall SF in diagnosing KD are shown in Figure 2. Due to significant heterogeneity of sensitivity (*I*^2^ = 84.81%, 95% CI: 79.05–90.58%, *P* < 0.001) and specificity (*I*^2^ = 92.55%, 95% CI: 90.26–94.85%, *P* < 0.001), the random-effects model was used. The pooled outcomes of the included diagnostic studies were as follows: sensitivity, 0.76 (95% CI: 0.69–0.82); specificity, 0.82 (95% CI: 0.76–0.88); PLR, 3.90 (95% CI: 2.87–5.30); NLR, 0.29 (95% CI: 0.22–0.38); and DOR 15.0 (95% CI: 9.00–25.00) (Supplementary Figure 2 and Table 4).

**TABLE 4:** Subgroup analysis of diagnostic accuracy of overall SF for KD.

	No	SEN	SPE	PLR	NLR	DOR	AUC	Heterogeneity	
		(95%CI)	(95%CI)	(95%CI)	(95%CI)	(95%CI)	(95%CI)	*I* ^2^	*P*-value
Overall	20	0.76 (0.69–0.82)	0.82 (0.76–0.88)	4.33 (3.07–6.11)	0.29 (0.22–0.38)	15.0 (9.00–25.00)	0.86 (0.83–0.89)	81.2%	0.000
Subgroup									
KD vs. all controls	11	0.79 (0.72–0.84)	0.86 (0.79–0.91)	4.61 (3.27–6.51)	0.26 (0.20–0.34)	20.82 (11.83–36.64)	0.89 (0.86–0.91)	73.0%	0.000
KD vs. healthy	3	0.72 (0.66–0.78)	0.77 (0.69–0.84)	3.04 (1.90–4.87)	0.37(0.29–0.47)	8.65 (4.37–17.14)	–	43.2%	0.172
KD vs. fever	2	0.80 (0.72–0.86)	0.82 (0.72–0.99)	7.66 (0.47–124.27)	0.24 (0.15–0.41)	30.32 (1.88–488.79)	–	84.1%	0.012
KD vs. s-JIA	6	0.82 (0.79–0.85)	0.87 (0.83–0.90)	5.72 (3.89–8.40)	0.22 (0.14–0.35)	31.93 (18.02–56.58)	0.92 (0.89–0.94)	53.1%	0.058
CAL vs. non-CAL	5	0.70 (0.70–0.70)	0.78 (0.78–0.78)	0.23 (0.09–0.56)	10.05 (3.10–32.59)	0.02 (0.00–0.11)	0.79 (0.75–0.82)	42.0%	0.136
IVIG responders vs. IVIG resistance	4	0.74 (0.49–0.90)	0.78 (0.65–0.87)	3.02 (1.77–5.14)	0.34 (0.14–0.82)	9.40 (2.70–32.72)	0.83 (0.79–0.86)	85.3%	0.000

KD, Kawasaki disease; IVIG, intravenous immunoglobulin; CALs, coronary artery lesions; s-JIA, systemic juvenile idiopathic arthritis; SEN, sensitivity; SPE, specificity; PLR, positive likelihood ratio; NLR, negative likelihood ratio; DOR, diagnostic odds ratio; AUC, area under the receiver operating characteristic curve; NA, not available.

#### Heterogeneity and subgroup analysis

**FIGURE 3:**
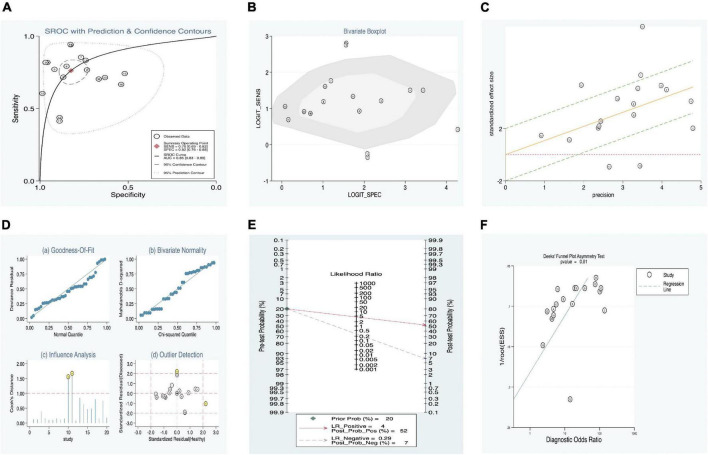
Estimation of diagnostic accuracy of overall SF in KD. **(A)** Summary receiver operator characteristic (SROC) curve; **(B)** bivariate boxplot; **(C)** Galbraith plot; **(D)** Cook’s distance plot; **(E)** Fagan’s nomogram; **(F)** Deek’s funnel plot.

In addition, a summary receiver operator characteristic (SROC) curve (Figure 3A) was utilized in this meta-analysis of KD vs. overall studies of SF, and the value of AUC (0.86, 95% CI: 0.83–0.89) was computed. Figure 3A suggests that no typical shoulder arm exists, thereby proving the non-attendance of the threshold effect. Spearman’s correlation coefficient value was –0.059, and the *P*-value was 0.805, demonstrating that there was no threshold effect. It could also demonstrate the fact that the threshold effect is not a source of heterogeneity. Although the bivariate boxplot and Galbraith plot are shown in Figures 3B,C, respectively, five studies fell outside the boxplot and Galbraith plot (30, 32, 33). Of these five studies, two studies were based on estimation between KD with SF values vs. controls with s-JIA (32, 33) and might be based on the analytic data (sensitivity = 60.40% vs. specificity = 98.80%) (32); two studies were based on estimation between IVIG responders and IVIG resistance [*n* = 271 and *n* = 29, respectively (30); *n* = 161 and *n* = 67, respectively (33)]; and another study was based on estimation between CAL (*n* = 48) and non-CAL (*n* = 252). (32) Moreover, Cook’s distance plot was used for influence analysis (Figure 3D), suggesting that two studies had strong sensitivity (32, 33), and the other studies did not cause sensitivity of the arithmetic results. Overall, the results of this study are relatively stable, indicating that pattern should be the main cause of heterogeneity.

Our meta-regression analysis suggests that the sample sizes in the included studies may contribute to the cause of heterogeneity. We also performed a subgroup analysis according to specimens in the included studies. The outcomes are presented in Table 4. The pooled AUC between SF levels in KD vs. all controls (11 studies) was 0.89 (95% CI: 0.86–0.91), corresponding to a sensitivity of 0.79 (95% CI: 0.72–0.84) and specificity of 0.86 (95% CI: 0.79–0.91). In addition, among the KD vs. all control groups, we separated the patients into three subgroups: (1) KD vs. healthy (three studies), (2) KD vs. fever (two studies), and (3) KD vs. s-JIA (six studies). Their pooled sensitivities vs. specificities were 0.72 vs. 0.77, 0.80 vs. 0.82, and 0.82 vs. 0.87, respectively. The subgroup of IVIG responders vs. IVIG resistance presented better diagnostic accuracy than that of the subgroup of CAL vs. non-CAL, with AUC values of 0.83 (0.79–0.86) and 0.79 (0.75–0.82) and with pooled DOR values of 9.40 (2.70–32.72) and 0.02 (0.00–0.11), respectively (Table 4).

Fagan’s nomogram was applied to evaluate the posttest probabilities, and the related result is shown in Figure 3E. We found that when 20% was chosen as the pretest probability, the posttest probability of SF was 52% of LR-positive and 7% of LR-negative.

Deek’s funnel plot asymmetry test was considered a useful tool for potential publication bias of diagnostic studies. The results showed that there was no significant publication bias, with a *P*-value of 0.01 (Figure 3F).

#### Meta-analysis serum ferritin levels of Kawasaki disease patients vs. controls

**FIGURE 4:**
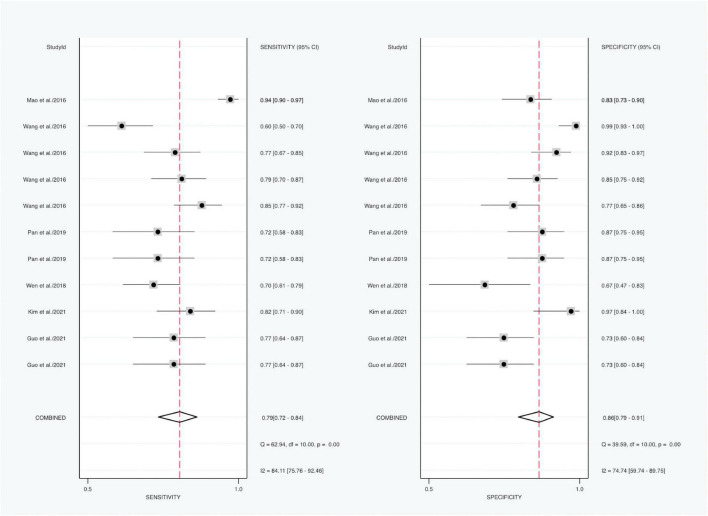
Forest plots of sensitivity and specificity of SF in the diagnosis of KD (KD vs. controls).

Forest plot results of related data from the 11 studies (six articles) (21, 28, 29, 31–33) on the sensitivity and specificity of SF in diagnosing KD are shown in Figure 4. Due to significant heterogeneity of sensitivity (*I*^2^ = 84.11%, 95% CI: 75.76–92.46%, *P* < 0.001) and specificity (*I*^2^ = 74.74%, 95% CI: 59.74–89.75%, *P* < 0.001), the random-effects model was used. The pooled outcomes of the included diagnostic studies were as follows: sensitivity, 0.79 (95% CI: 0.72–0.84); specificity, 0.86 (95% CI: 0.79–0.91); PLR, 4.61 (95% CI: 3.27–6.51); NLR, 0.26 (95% CI: 0.20–0.34); and DOR 20.82 (95% CI: 11.83–36.64) (Supplementary Figure 3).

#### Meta-analysis serum ferritin levels of Kawasaki disease patients versus controls

**FIGURE 5:**
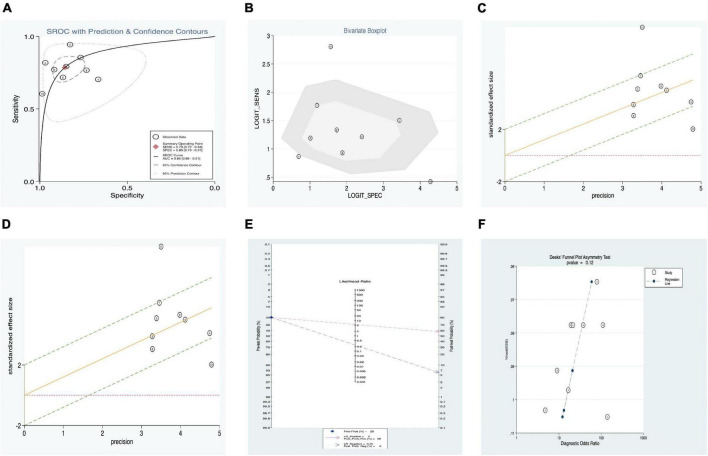
Estimation of diagnostic accuracy of SF vs. control (fever, healthy, and s-JIA) in KD. **(A)** Summary receiver operator characteristic (SROC) curve; **(B)** bivariate boxplot; **(C)** Galbraith plot; **(D)** Cook’s distance plot; **(E)** Fagan’s nomogram; **(F)** Deek’s funnel plot.

In addition, a summary receiver operator characteristic (SROC) curve was utilized in this meta-analysis of SF levels of KD patients vs. controls (Figure 5A) and computed the AUC value (0.89, 95% CI: 0.86–0.91). Figure 5A suggests that no typical shoulder arm exists, thereby proving the non-attendance of the threshold effect. Spearman’s correlation coefficient value was 0.064, and the *P*-value was 0.851, thereby demonstrating that there was no threshold effect. Furthermore, it could also demonstrate the fact that the threshold effect is not a source of heterogeneity. Although the bivariate boxplot and Galbraith plot are shown in Figures 5B,C, three studies fell outside the boxplot and Galbraith plot (31–33). Of the three studies, the sample size of one study was 108 KD patients vs. 30 healthy controls (31), the sample size of the second study was 228 KD patients vs. 81 s-JIA controls (33), and the third study was based on sensitivity (60.40%) vs. specificity (98.80%) (32). Moreover, Cook’s distance plot was used for influence analysis (Figure 5D), indicating that the pattern should be the cause of heterogeneity.

Fagan’s nomogram was applied to evaluate the posttest probabilities, and the related result is shown in Figure 5E. We found that when 20% was chosen as the pretest probability, the posttest probability of SF was 58% LR-positive and 6% LR-negative.

Deek’s funnel plot asymmetry test was considered a useful tool for potential publication bias of diagnostic studies. The results showed that there was no significant publication bias, with a *P*-value of 0.12 (Figure 5F).

### Prognostic value of serum ferritin for Kawasaki disease

#### Study characteristics and quality assessment

A total of 894 patients were included from five studies (four articles) (33–36) relevant to SF levels in KD. A total of four studies examined the relationship between the SF level and IVIG responders vs. IVIG-resistant groups, and the fifth study examined the relationship between the SF level and CAL vs. non-CAL groups. One study contained two ORs and 95% CI values [the first value was from patients who did not need plasma exchange (PE), and the second value was from patients who needed PE] (33). The sample sizes of the included studies ranged from 92 to 228, and the prognostic studies were published from 2016 to 2021. The detailed characteristics of each included study and NOS score are shown in Supplementary Table 1. All OR values were obtained directly from publications. The baseline characteristics of all included studies are summarized in Table 3.

#### Data analysis

**FIGURE 6:**
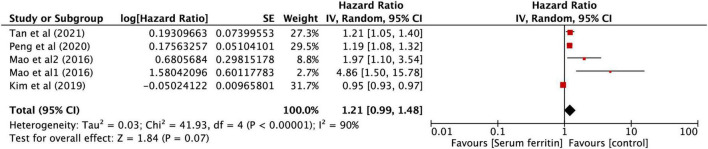
Forest plots of hazard ratios for the association between expression of SF for KD in five studies.

**FIGURE 7:**
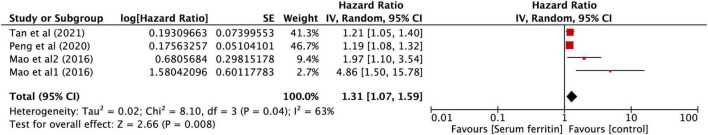
Forest plots of hazard ratios for the association between expression of SF for KD in four studies.

After performing the heterogeneity test on five studies, we obtained the following results: χ^2^ = 41.93, df = 4, *I*^2^ = 90% > 50%, and *P* = 0.0001 < 0.1 (random-effects model) in the *Q*-test, indicating that there was strong significant publication bias between the five studies. The pooled hazard ratio (HR) of the five records reached 1.21 (95% CI: 0.99, 1.48), and the results were significant (*Z* = 1.84, *P* = 0.07 > 0.05), indicating that the risk in SF may contribute to the development of KD in children (Figure 6). However, the literature has a great influence on the results, which indicated that the results of this study are not relevantly steady (Supplementary Figures 4A,B). Kim’s study may have a particular impact on the stability of the results, and after withdrawing Kim’s study (34), the remaining four studies obtained better results. The related outcomes were as follows: χ^2^ = 8.10, df = 3, *I*^2^ = 63% > 50%, and *P* = 0.04 < 0.1 (random-effects model) in the *Q*-test, demonstrating that there was significant publication bias between the four records. The pooled HR of the four records reached 1.31 (95% CI: 1.07, 1.59), and the results were significant (*Z* = 2.66, *P* = 0.008 > 0.05), illustrating that the risk in SF could contribute to the development of KD in children (Figure 7). The sensitivity analysis of the study is shown for SF and illustrated that the results of this study are relatively stable (Supplementary Figure 6A). However, Begg’s test results (*P* = 0.09 > 0.05) and Egger’s test results (*P* = 0.015 < 0.05) based on the funnel plot (Supplementary Figure 5B) were still within the acceptable range.

## Discussion

Not only is ferritin a ubiquitous intracellular protein characterized by storing iron, but it is also an acute-phase reactant widely utilized in clinical practice (37, 38). Under inflammatory conditions, ferritin synthesis is markedly induced by TNF-α and IL-1α, which are released from activated macrophages (39, 40). Emerging research has proven that serum ferritin levels are elevated in patients with certain inflammatory conditions, including rheumatoid arthritis, systemic lupus erythematosus, chronic kidney disease, COVID-19 caused by the virus SARS-CoV-2, thyroiditis, and others. (41–45). Meanwhile, an increasing number of studies have shown that SF is aberrantly elevated in KD and related complications (20, 21, 39, 40), and hyperferritinemia (i.e., a ferritin level above 500 mg/L) is considered one of the diagnostic criteria in HLH-2004 (41). More interestingly, with the deepening research on ferroptosis, we realized that ferroptosis, a new type of programmed cell death, plays a crucial role in the pathophysiological process of various diseases (19, 38), and ferritin might be an important switch in cell ferroptosis (18). All signs indicate that SF seems to be a good prospective ideal biomarker for the diagnosis or prognosis of IVIG resistance and CALs in KD when compared to other biomarkers. Therefore, our study conducted a comprehensive and systematic meta-analysis to assess the relationship between the SF level and diagnostic and prognostic efficiency in IVIG resistance and CALs in KD based on 3506 patients.

Our study found that SF has relatively high diagnostic efficiency in discriminating KD patients from overall controls. In general, the minimum value of DOR is 0, and the maximum value is infinity; a higher value of DOR suggests a better discriminatory test performance (46). All these results suggested that SF might be suitable as a potential biomarker for diagnosing KD, which is consistent with conclusions from other studies (20, 47). Ferritin is an acute-phase reactant that is utilized in clinical practice as a serum biomarker (37), which regulates the homeostasis of serum iron, and elevated ferritin concentration signifies high levels of stored iron (48). Excessive free iron induces cell ferroptosis characterized by the iron-dependent accumulation of lipid ROS and leads to cytological changes (49); growing evidence has proven that activating or blocking the ferroptosis pathway alleviates the progression of the disease, which provides a promising therapeutic strategy for many diseases (17, 19).

Generally, KD has a good prognosis, and clinical symptoms improve if diagnosed and treated early. However, if the diagnosis or treatment is not made in time, CAL sequelae or even death may occur, with an approximately 0.02–1% mortality rate, especially when combined with IVIG-resistant patients (1, 50, 51). However, IVIG resistance and CALs adversely cause life-threatening conditions in pediatric KD (1, 52). Numerous studies have investigated CALs as a more common occurrence of approximately 15–25% in KD (1, 52, 53). However, the specific factors or main causes of developing CALs in childhood KD remain unclear. Recent studies of KD related to IVIG resistance and CALs in children may be the causes of SF levels (20, 21, 28–33). Moreover, elevated SF is a biomarker of IVIG resistance and CALs in KD, as reported by Kong et al. (30). In addition, the survey by Kim et al. (21) also shows that SF may be a diagnostic biomarker for KD. The aforementioned results and our meta-analysis confirmed that SF may be used as a workable and critical biomarker for the diagnosis of patients with KD and IVIG resistance. Although our subgroup analysis based on CALs vs. non-CALs presented a poorer diagnostic accuracy than that based on IVIG responders vs. IVIG resistance, its corresponding sensitivity and specificity values were still greater than 0.70. A series of current published studies from 2015 to 2021 investigated the SF level as a useful marker for the prediction of resistance to initial IVIG therapy (20, 30, 31, 33). Our group considered that SF may still be a potential biomarker to predict CALs in KD with sufficient research. To the best of our knowledge, our meta-analysis is the first to assess the diagnostic roles of SF in KD, IVIG resistance, and CALs and innovatively proposes that maintaining the dynamic balance between iron, SF, and ferroptosis will be an important therapeutic strategy to reduce morbidity in pediatric acquired heart disease. Moreover, it is a known fact that SF could be elevated in KD (with abundantly available research). The issue occurs when one tries to make a cutoff value that predicts IVIG resistance and that predicts CALs. Various RCTs propose various cutoffs regarding the same. In that situation, it is difficult for the physician to rely on a single value. Still, this meta-analysis could add to the available research.

Although numerous studies have investigated KD continuously, the particular risk factors for SF in developing childhood KD remain unknown (33–36). Elevated serum ferritin levels are significantly related to IVIG resistance in KD (30, 54). Moreover, SF levels are notably increased in KD with CALs (30, 33, 34). In our meta-analysis, we found that SF was vital to the prognosis of IVIG resistance and CALs in KD. To our knowledge, our article is the first meta-analysis of the relationship between SF and the prognostic roles of IVIG resistance and CALs in KD. The aggregated results confirmed that SF may be a potential prognostic biomarker for IVIG resistance and CALs in KD.

### Strength and limitations

Most of the included studies are systematic reviews to conduct the biomarker in the diagnosis and prognosis associated with SF in pediatric KD. To our knowledge, this is by far the most comprehensive research on the biomarker in the diagnosis and prognosis associated with SF in pediatric KD by using the meta-analysis. Nevertheless, there are still several limitations to our current meta-analysis. First, the number of included studies was small (*n* = 11), and all of the studies were conducted in Asian populations (China, Korea, and Japan). There were no data from other countries or regions. Thus, our conclusions are only applicable to Asian populations, which may affect the external applicability of our results in different regions. However, according to the recent global epidemiology of vasculitis, Kawasaki disease occurs most frequently in East Asia, especially Japan, South Korea, and China, with a relatively equal distribution elsewhere (2). Second, all of the follow-up times of the included articles were unclear, which may lead to the deviation of CAL diagnosis and could further affect the accuracy of FS in predicting CALs of KD. Third, although we conducted hierarchical analyses, heterogeneity still existed in some subgroups.

## Conclusion

In summary, this meta-analysis identified SF as a workable and critical biomarker for the diagnosis of KD patients, including the prediction of CALs and IVIG resistance in patients. In addition, our study demonstrated a significant association between SF and the prognosis of KD, signifying that SF may have an essential role in the occurrence and prognosis of KD, but we suggest multicenter (even multi-national) approaches or more powerful research in obtaining larger sample sizes to prove. We also propose that maintaining the dynamic balance between iron, SF, and ferroptosis will be an important therapeutic strategy to reduce morbidity in pediatric acquired heart disease. However, more comprehensive studies with large scale, more regions, and high quality should be performed to elucidate the roles of SF in diagnosing and predicting IVIG resistance and CALs in KD.

## Data availability statement

All datasets generated for this study are included in the article/Supplementary material.

## Author contributions

HW and MH conceived the idea for the study. HW, MH, and MZ selected studies for inclusion and abstracted data. MH and PH conducted the statistical analyses. MH and HW interpreted the data. HW and MH wrote the first draft. MZ and QH critically revised the manuscript for important intellectual content. All authors have read and approved the content of the manuscript.

## Footnotes

1
http://www.cochrane.org

2
http://www.hrc.es/investigacion/metadisc_en.htm

3
https://www.stata.com
