# Supplementary material for: Serum ferritin as a crucial biomarker in the diagnosis and prognosis of intravenous immunoglobulin resistance and coronary artery lesions in Kawasaki disease: A systematic review and meta-analysis
Source: Front Med (Lausanne). 2022 Aug 10;9:941739. doi: 10.3389/fmed.2022.941739 (PMC9399505; doi:10.3389/fmed.2022.941739)
Supplement: Supplementary file 2 [file Table_2.docx]

| Author | Selection | | | | comparability | Outcome | | | NOS |
| --- | --- | --- | --- | --- | --- | --- | --- | --- | --- |
|  | Representativeness  Of the exposed | Selection of the  non-exposed  cohort | Ascertainment  Of expose | Demonstration that outcome of interest was not present at start of study | Comparability of  Cohort on the basis of the design or analysis | Assessment of outcome | Was follow-up long enough for outomes | Adequency of follow-up  Of cohorts | Overall score |
| Tan et al (2021) | ☆ | ☆ | ☆ | ☆ | ☆☆ | ☆ | ☆ | ☆ | 9 stars |
| Peng et al (2020) | ☆ | - | ☆ | ☆ | ☆☆ | ☆ | ☆ | ☆ | 8 stars |
| Kim et al (2019) | - | ☆ | ☆ | ☆ | ☆☆ | ☆ | ☆ | ☆ | 8 stars |
| Mao et al (2016) | ☆ | ☆ | ☆ | ☆ | ☆☆ | ☆ | ☆ | ☆ | 9 stars |

**Supplementary Table 1:** Quality scores of included studies using newcastle-ottawa scale

Note:the NOS scale full score is nine stars; one–three stars are low-quality study, four–six stars are medium-quality study, and seven–nine stars are high-quality study.
