# Supplementary material for: Serum ferritin as a crucial biomarker in the diagnosis and prognosis of intravenous immunoglobulin resistance and coronary artery lesions in Kawasaki disease: A systematic review and meta-analysis
Source: Front Med (Lausanne). 2022 Aug 10;9:941739. doi: 10.3389/fmed.2022.941739 (PMC9399505; doi:10.3389/fmed.2022.941739)
Supplement: Supplementary file 4 [file Table_4.DOCX]

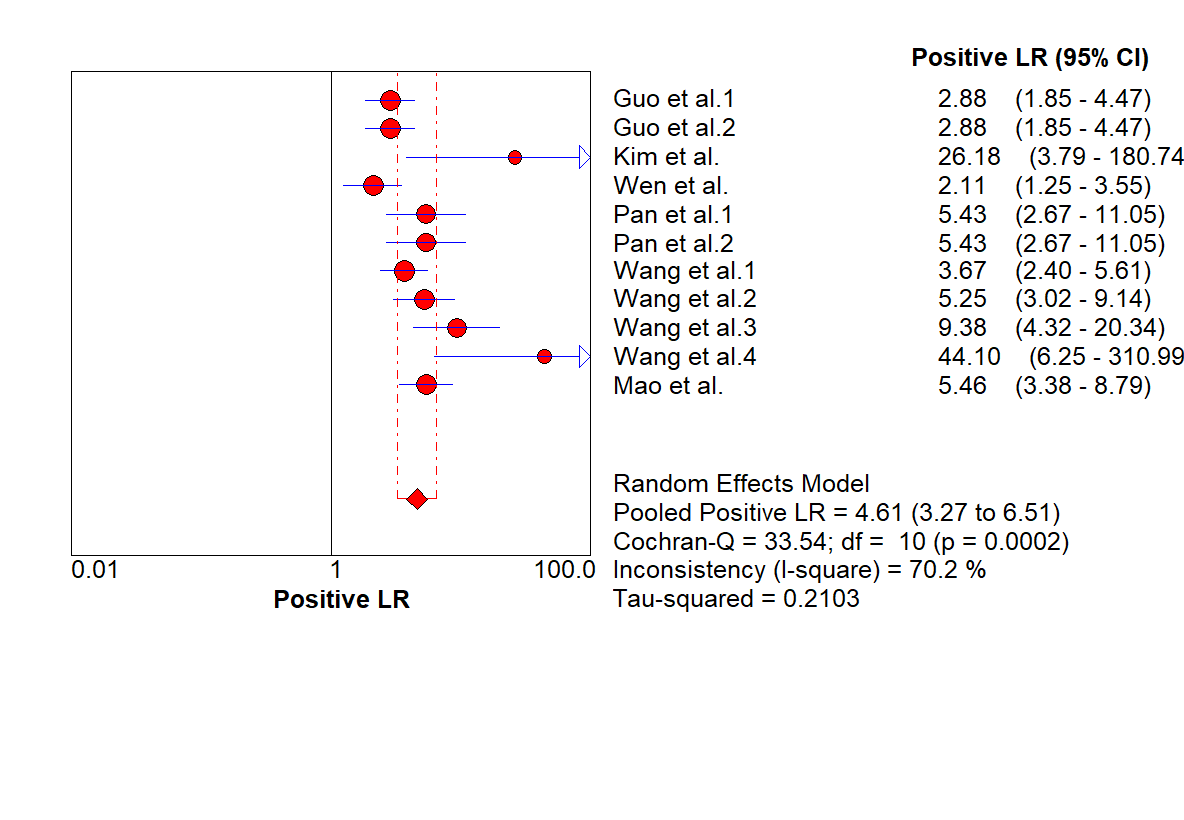

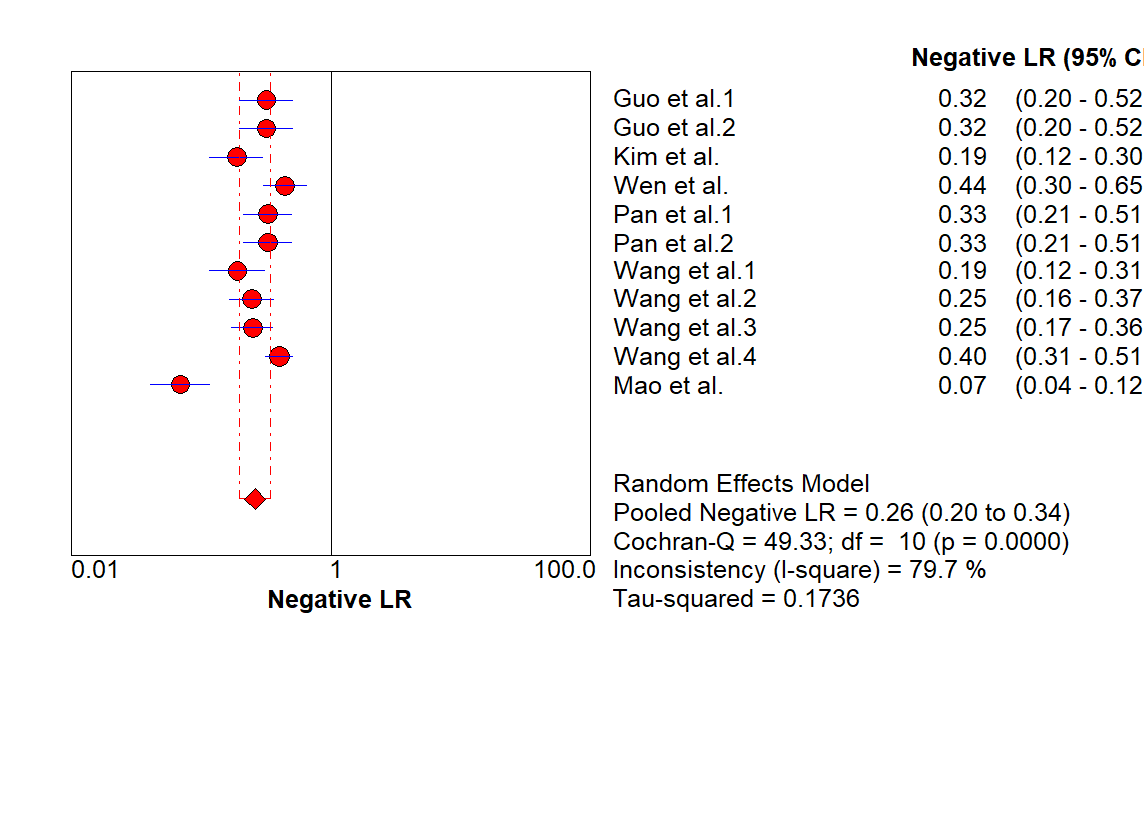

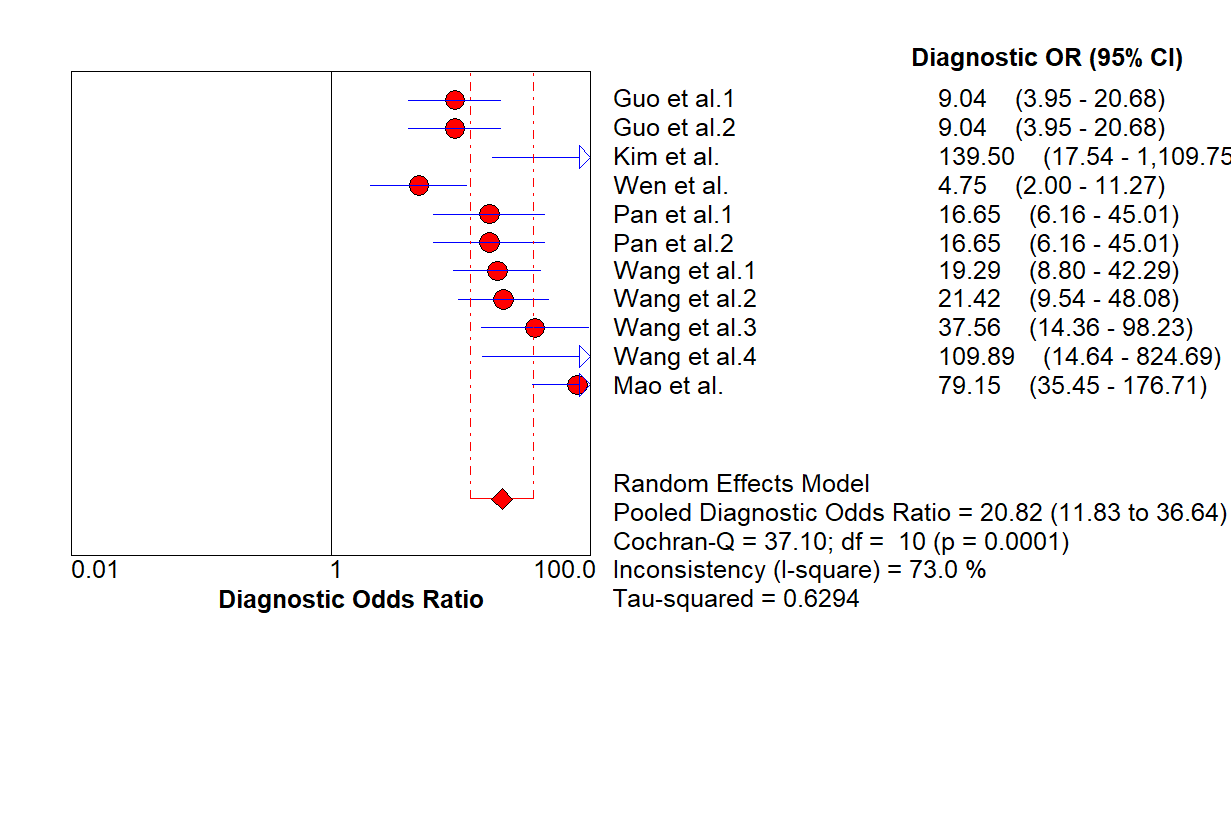


Supplementary Figure 4. positive likelihood ratio, negative likelihood ratio, and diagnostic odds ratio from the 11 studies (6 articles)
