# Supplementary material for: Serum ferritin as a crucial biomarker in the diagnosis and prognosis of intravenous immunoglobulin resistance and coronary artery lesions in Kawasaki disease: A systematic review and meta-analysis
Source: Front Med (Lausanne). 2022 Aug 10;9:941739. doi: 10.3389/fmed.2022.941739 (PMC9399505; doi:10.3389/fmed.2022.941739)
Supplement: Supplementary file 5 [file Table_5.DOCX]

Supplementary Figure 5A. The sensitivity analysis of 5 studies


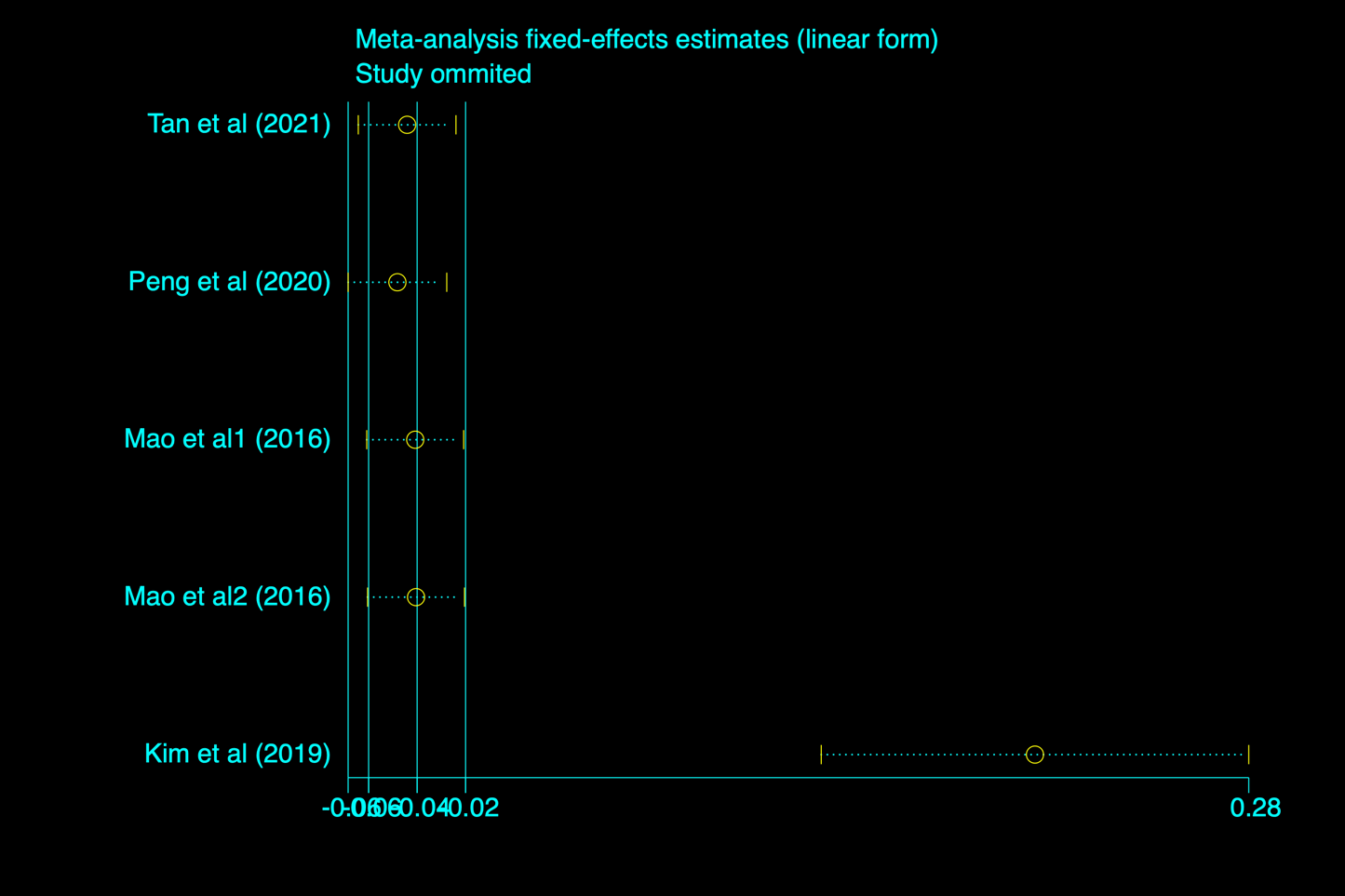


Supplementary Figure 5B. Funnel plot of 5 studies
