# Supplementary material for: Serum ferritin as a crucial biomarker in the diagnosis and prognosis of intravenous immunoglobulin resistance and coronary artery lesions in Kawasaki disease: A systematic review and meta-analysis
Source: Front Med (Lausanne). 2022 Aug 10;9:941739. doi: 10.3389/fmed.2022.941739 (PMC9399505; doi:10.3389/fmed.2022.941739)

Supplementary Figure 6A. The sensitivity analysis of 4 studies


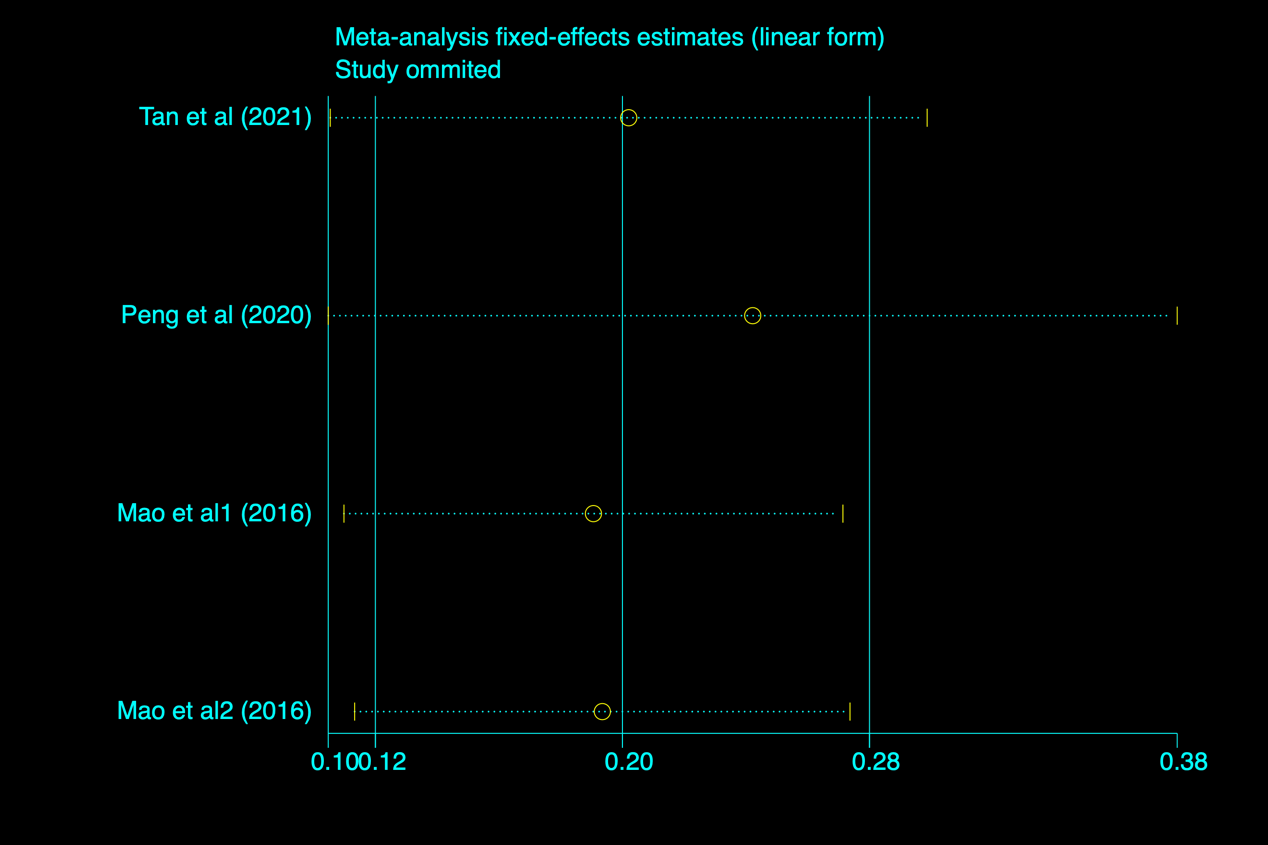


Supplementary Figure 6B. Funnel plot of 4 studies


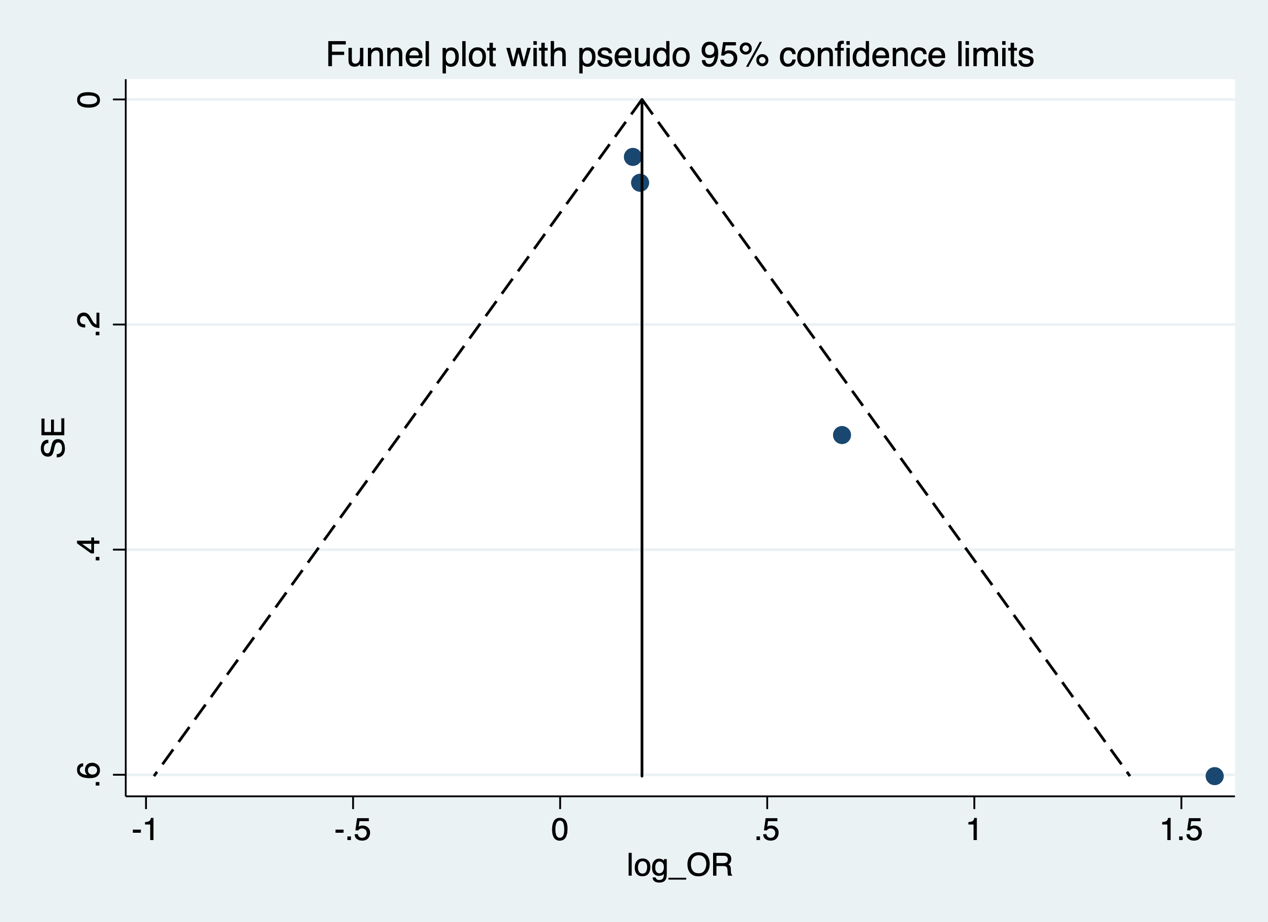

Supplement: Supplementary file 6 [file Table_6.DOCX]
